# Age‐Matched Reference Values for Circulating Natural Killer T (NKT)‐Like Cells

**DOI:** 10.1111/sji.70062

**Published:** 2025-10-26

**Authors:** Elena Trombetta, Manuela Liguori, Federico Simone Colombo, Marta Tornese, Alessandra Cattaneo, Daniele Prati, Ferruccio Ceriotti, Laura Porretti

**Affiliations:** ^1^ Flow Cytometry Laboratory, Clinical Pathology, Fondazione IRCCS ca' Granda Ospedale Maggiore Policlinico Milan Italy; ^2^ Transfusion Medicine Department, Fondazione IRCCS ca' Granda Ospedale Maggiore Policlinico Milan Italy

**Keywords:** lymphocytes, NKT‐like cells, reference values

## Abstract

Natural Killer T‐like cells are T lymphocytes characterised by the expression of CD56 accompanied by perforin and granzyme B production, typical of cytotoxic cells and the innate immunity compartment. Imbalance in their distribution could occur in pathological conditions, including solid or haematological cancers, autoimmune disorders and metabolic diseases; therefore we aimed to define age‐matched reference values of the NKT‐like population. NKT‐like cell count and their expression of CD4 and CD8 surface antigens were evaluated in 2208 subjects by flow cytometry, with a lyse‐no‐wash protocol in a CE‐IVD workup. Outpatients without alteration of any laboratory test and adult healthy volunteers were used to determine NKT‐like age‐matched reference values; a total of 490 subjects divided into six age ranges were finally enrolled. We defined reference values for NKT‐like cells in adult and in 5 age‐matched paediatric control groups. NKT‐like cell number increases with age both in percentage and absolute count. While NKT‐like/CD8+ cells increased with age, NKT‐like/CD4+ cells showed an opposite trend. Similarly, double‐positive cells (NKT‐like/CD4+ CD8+) gradually decreased with age, while double‐negative cells (NKT‐like/CD4‐CD8‐) increased up to 16 years and then decreased in adults. Ultimately, we evaluate adult and paediatric patients with NKT‐like cell count outside normal ranges derived here to highlight the principal associated conditions that may be involved. Our paper is a first step toward the definition of robust reference values for NKT‐like cell subpopulations under the CE‐IVD conditions. Providing NKT‐like cell number and frequency in the diagnostic report appears helpful to better delineate the immune profile of patients.

## Graphical abstract

NKT‐like lymphocyte reference values defined in over 300 children, across five age groups, via CE‐IVD immunophenotyping. Provide a benchmark for paediatric immune monitoring supporting clinical decisions in routine laboratory practice.

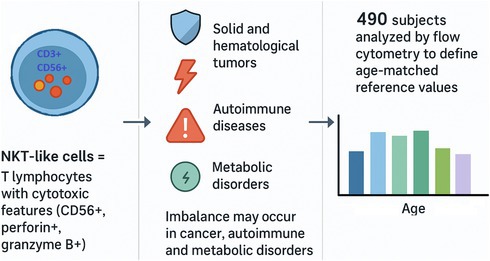

**Abbreviations**
APC: allophycocyanin
CD: cluster of differentiation
CE‐IVD: Conformité Européenne—In Vitro Diagnostic
CS&T: cytometer setup and tracking
Cy: cyanine
EDTA: ethylenediaminetetraacetic acid
FACS: fluorescence activated cell sorting
FITC: fluorescein isothiocyanate
HD: healthy donors
IQR: interquartile range
MAIT: mucosal‐associated invariant T
NCAM: neural cell adhesion molecule
NKT: natural killer T
PE: R‐phycoerythrin
PerCP: peridinin‐chlorophyll‐protein complex
ROUT: robust regression and outlier removal
SD: standard deviation
TCR: T‐cell receptor

## 1 Introduction

Natural Killer T (NKT)‐like cells are a heterogeneous population of T lymphocytes characterised by the surface expression of the neural cell adhesion molecule (NCAM, cluster of differentiation CD56) [1]. Even if CD56 was initially identified in the neural system, and is thus considered a neural lineage marker, its expression can also be found in the immune system, in particular as distinctive of, but not restrictive to, NK cells [2]. Expression of CD56 on T‐cells correlates with the ability to produce and store perforin and granzyme B, cytolytic molecules typical of cytotoxic and first‐line defence cells, such as the innate immunity compartment [3, 4]. Although CD56 expression on T lymphocytes is often considered a hallmark of activation, it is more accurately associated with effector functions and limited proliferative capacity, the latter being a feature typically linked to senescent cells [5, 6].

Beyond CD56, NKT‐like cells can also express other cytotoxic receptors characteristic of the NK compartment, such as NKp44 and NKp46, as well as other classic markers of activation, typically CD69 and HLA‐DR [7].

The nomenclature of CD56‐positive T lymphocyte subpopulations is controversial due to the existence of other T‐cell subpopulations expressing NK antigens, and thus defined as NKT cells. There is growing evidence that this cell compartment comprises both the unconventional and conventional T lymphocytes. Among them are found invariant NKT (iNKT) cells (accounting for 0.3%–0.5% of total T lymphocytes in adults and 0.08%–0.7% in children) and the mucosal‐associated invariant T (MAIT) cells (up to 10% of CD8+ or CD4− CD8− peripheral T lymphocytes). The first are characterised by TCR restriction expressing the variable (V) and joining (J) Vα24Jα18 chain, conferring them the ability to recognise the sphingolipid alpha‐Galactosylceramide presented by the CD1d MHC‐like molecule; the latter are restricted to the MHC‐I‐like molecule MR1, express an invariant TCRα chain (TRAV1/TRAJ33), and are involved in the defence against microbial infection [8, 9, 10, 11, 12, 13, 14]. To better dissect the composition of this population, Romero‐Olmedo and colleagues recently provided a detailed phenotype description of different subpopulations of NKT‐like cells, recognising about 19 cell clusters, characterised by different cytotoxic potentials and belonging either to innate or classic T‐cell immunity [15]. From the first group it can be found the above‐mentioned iNKT and MAIT, and a fraction of TCRγδ lymphocytes, which could express CD56 antigen [16, 17]. The second group included cells from either T helper (Th) 1, −2 and −17 subsets [15].

Imbalances in NKT‐like cell distribution can be detected in several pathologic conditions, including solid or haematological cancers, autoimmune disorders and metabolic diseases, such as Type 1 and 2 diabetes [18, 19, 20, 21, 22, 23, 24, 25]. NKT‐like cells are also involved in viral infections and chronic lung disorders and have been recently investigated in SARS‐CoV‐2 infection, where cell count was significantly reduced in patients compared with healthy subjects and correlated with disease severity and PaO2/FiO2 ratio [26, 27, 28, 29, 30, 31].

Few data are so far available regarding the normal values of circulating NKT concentration, especially in the paediatric population. Therefore, in this study we defined narrow age range reference intervals of peripheral NKT‐like cells from routinely performed lymphocyte immunophenotyping, referring to the interval between the 5th and the 95th percentile.

## 2 Materials and Methods

### 2.1 Subjects

We retrospectively analysed NKT‐like cell counts from lymphocyte immunophenotyping, performed on 2208 outpatients referred to our hospital, from May 2019 to March 2020. Patients with alterations in laboratory findings, a history of immunological or haematological diseases, immunosuppressive therapies, or ongoing infections were excluded. We finally retrieved 449 subjects divided into six age groups, according to the age intervals routinely used in our laboratory, defined by Comans‐Bitter [32]: 0–15 months, 15–24 months, 2–5 years, 5–10 years, 10–16 years, and over 16 years. NKT‐like cell count was also evaluated on left‐over EDTA blood samples of 41 healthy blood donors (healthy donors, HD) after their informed consent was obtained. In order to increase the number of paediatric subjects within each age group, we also included all the analyses performed on paediatric patients until August 2021.

After defining reference values for NKT‐like cells from the derivation cohort, we stratified adult and paediatric patients, excluded from the derivation cohort according to the criteria reported above, identifying 532 subjects with either lower or higher NKT‐like cell counts compared to the established normal ranges.

The study was approved by the Institutional Review Board Lombardia 3 (#358_2020) and was conducted in accordance with the Helsinki Declaration.

### 2.2 Flow Cytometry Analysis

Lymphocyte immunophenotyping was carried out using Multitest 6‐colour TBNK Kit (Cat. 337,166, BD Biosciences, San Jose, CA), a cocktail of seven fluorochrome‐conjugated monoclonal antibodies consisting of CD3‐FITC (clone SK7), CD16 and CD56‐PE (clones B73.1 and NCAM16.2), CD45‐PerCP‐Cy5.5 (clone 2D1), CD4‐PE‐Cy7 (clone SK3), CD19‐APC (clone SJ25C1), CD8‐APC‐Cy7 (clone SK1). Trucount Tubes (BD) were used to obtain absolute cell counts, following the standard procedures for the Lyse‐No‐Wash protocol. Samples were processed within 4 h from blood withdrawal, acquired with a FACSLyric, and analysed using the dedicated template with FACSuite Clinical Software 1.4 (all from BD). Instrument calibration and spillover matrix were updated on a daily basis using the Cytometer Setup and Tracking (CS&T) beads (Cat. 656,505, BD). The entire diagnostic protocol and reagents were under the CE‐IVD certification. The gating strategy was depicted in Figure S1A; NKT‐like cells were defined as lymphocytes expressing CD3/CD16 and CD56 antigens. To verify whether this population effectively expressed CD56, CD16 or both, we randomly selected 30 subjects during the study to perform an additional three‐colour panel, composed of CD56‐FITC (NCAM16.2), CD3‐PE (clone SK7), and CD16‐PE‐Cy7 (clone B73.1, Figure S1B). Expression of CD4, CD8 or negativity for both these T‐cell markers was also evaluated on the NKT‐like cell population.

### 2.3 Statistical Analysis

GraphPad Prism 9.2 (GraphPad Software, San Diego, CA) was used to identify and remove outliers (*n* = 21) with the robust regression and outlier removal (ROUT) method. Age distributions were reported as mean and standard deviation (SD), while gender was reported as a percentage. For each group's descriptive statistics, linear regression, median, and 5th and 95th percentiles of both NKT‐like cell percentages and absolute counts were obtained. Expression of CD4+ and CD8+ on NKT‐like cells was reported as median and interquartile range (IQR). Since only the HD group showed a normal distribution (Kolmogorov–Smirnov test), data were analysed with a non‐parametric test (Mann–Whitney *U* test).

## 3 Results

A total of 490 subjects divided into six age ranges were finally enrolled: 44 (0–15 months), 72 (15–24 months), 85 (2–5 years), 65 (5–10 years), 66 (10–16 years), 117 (> 16 years) and 41 blood donors (age 18–65 years) (Table 1). Since NKT‐like cell counts obtained in healthy donors were not statistically different from those observed in adult patients (HD = 111/μL vs. > 16 years grou*p* = 91/μL, *p* = 0.25), we merged these two groups together.

**TABLE 1:** Reference values of absolute NKT‐like cell count (/μL) and percentage (%) expressed as median and 5th–95th percentiles.

Age ranges	0–15 months	15–24 months	2–5 years	5–10 years	10–16 years	> 16 years	HD	> 16 years + HD
*N* subjects	44	72	85	65	66	117	41	158
Age (mean, SD)	7.5 (4.6)	18.7 (2.7)	2.9 (0.8)	6.5 (1.3)	12.3 (1.8)	42.4 (17.4)	44.9 (13.8)	43.1 (16.5)
Sex	54.5% M 45.5% F	54.2% M 45.8% F	49.4% M 50.6% F	60% M 40% F	48.6% M 51.4% F	36.8% M 63.2% F	56.1% M 43.9% F	41.8% M 58.2% F
NKT‐like cells/μL	14 (3–52)	18 (3–48)	26 (10–68)	46 (12–114)	53 (15–153)	91 (28–223)	111 (14–252)	97 (27–225)
% NKT‐like cells on lymphocytes	0.2 (0.03–1.0)	0.3 (0.07–1.0)	0.7 (0.2–1.9)	1.5 (0.4–4.6)	2.4 (0.6–7.2)	4.7 (1.4–11.1)	6.7 (1.0–12.3)	4.8 (1.3–11.2)
% NKT‐like cells on CD3+ lymphocytes	0.7 (0.0–1.2)	0.6 (0.1–1.5)	1.0 (0.3–3.0)	2.2 (0.5–6.4)	3.4 (0.9–9.5)	6.2 (1.9–14.0)	8.3 (1.2–14.8)	6.5 (1.7–14.3)

**FIGURE 1:**
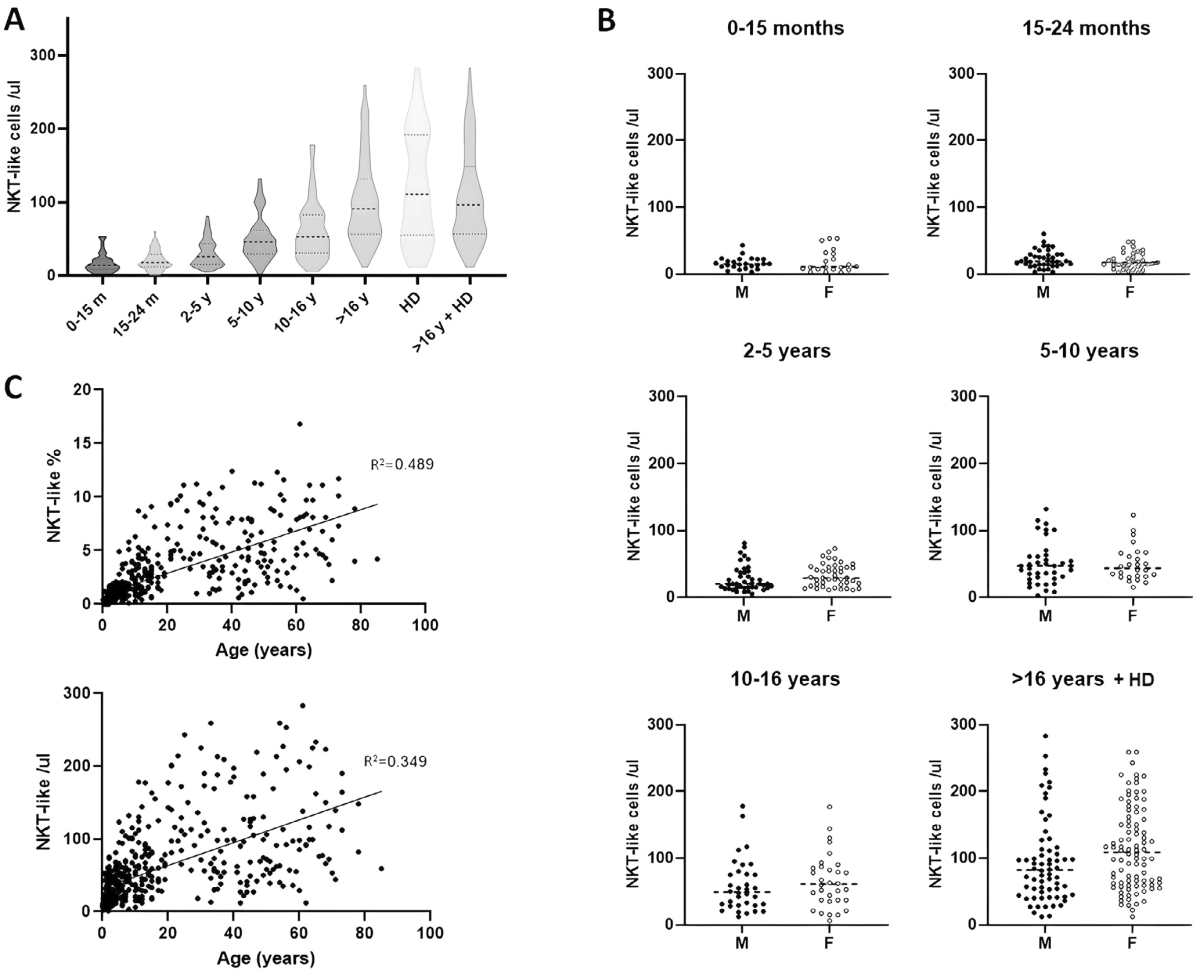
NKT‐like absolute count. (A) Violin plots showing the NKT‐like absolute cell count in each age group; medians are represented by dotted lines (HD, adult healthy volunteers; m, months; y, years). (B) Scatter dot plots were used to depict the distributions of NKT‐like absolute cell count in male (M) and female (F) subjects. Mann–Whitney *U* test was used to determine statistical differences between the two groups. (C) Simple linear regressions of NKT‐like cells with age, in percentages (top) and absolute counts (bottom).

For each age group, principal demographic data, NKT‐like cell counts and percentages are reported in Table 1 and Figure 1A. We did not detect a gender effect in any age group, even in the adult group, which was the richest in female subjects (58.2%), it can be noted that there is a higher NKT‐like cell count in females as compared to males (109/μL, 62–161, vs. 82/μL, 45–114, median and percentile range, Mann–Whitney *U* test, *p* = 0.057; Figure 1B).

**FIGURE 2:**
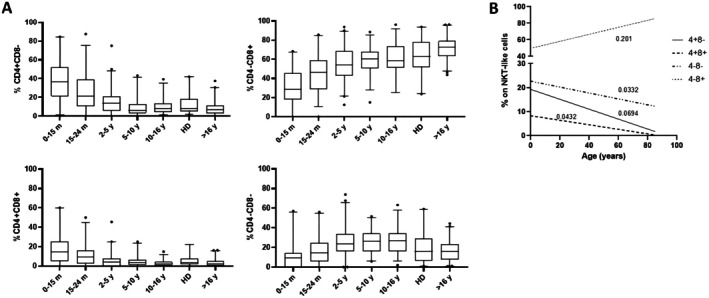
Expression of CD4 and CD8 antigens as a percentage of the NKT‐like cell population along with age distribution. (A) Box plots (HD, adult healthy volunteers; m, months; y, years) and (B) Simple linear regression analyses of NKT‐like cell subpopulations and age.

We also observed a trend in both percentages and absolute NKT‐like cell counts that increases with subjects' age, although not reaching statistical significance (simple linear regression, *R* ^2^ = 0.49 and *R* ^2^ = 0.35 for percentages and absolute counts, respectively; Figure 1C). Analysing the expression of CD4 and CD8 antigens on the NKT‐like cell population, we observed that these cells were predominantly CD8+. Interestingly, while NKT‐like/CD8+ cells increased with age, NKT‐like/CD4+ cells showed an opposite trend. Similarly, double‐positive cells (NKT‐like/CD4+ CD8+) gradually decreased with age, while double‐negative cells (NKT‐like/CD4− CD8−) increased up to 16 years and then decreased in adults (Figure 2A,B and Table S1).

The group of randomly selected subjects stained with the additional second panel (*n* = 30) allowed us to assess that the expression of CD3, detected from TBNK analysis, was almost completely due to CD56 antigen alone or, in a very small fraction, in combination with CD16; CD3 lymphocytes expressing CD16 alone were rarely observed (Figure S1B).

**FIGURE 3:**
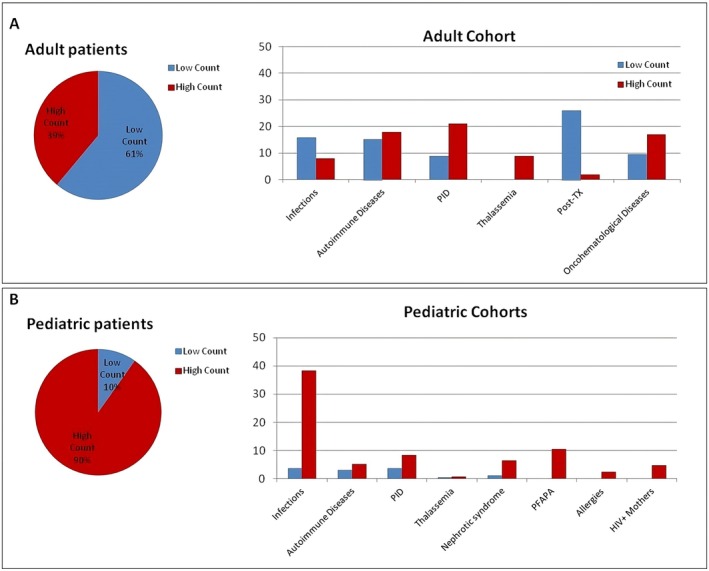
Out of range patients low or high NKT‐like proportion and pathological conditions. (A) Pie charts (left) and histograms (right) of the distribution of adult patients (%) with low (blue, *n* = 157) or high (red, *n* = 100) NKT‐like cells count, and their pathological condition; (B) Pie charts (left) and histograms (right) of the distribution of paediatric patients (%) with low (blue, *n* = 27) or high (red, *n* = 248) NKT‐like cells count, and their pathological condition.

Patients with NKT‐like cells outside the established reference values were divided into two groups: those with a low NKT‐like cell count (< 5th percentile, low count cohort), and those with a high count (> 95th percentile, high count cohort). In particular, for adult patients 157 (61%) subjects had a count lower than 27 cells/μL, and 100 (39%) had a count higher than 225 cells/μL (Figure 3A). Paediatric patients were firstly evaluated according to their appropriate age‐reference values, and then were grouped all together (0 months–16 years): the low cohort was composed of 27 (10%) patients, while the high count cohort consisted of 248 (90%) children (Figure 3B). Examining in more detail these patients, we grouped them into the most recurrent pathological conditions, such as infections (viral or bacterial), autoimmune diseases, primary immunodeficiencies (PID), thalassaemia, transplants (bone marrow or solid organs), oncohematological diseases, nephrotic syndrome, PFAPA (periodic fever, aphthous stomatitis, pharyngitis, and cervical adenopathies), allergies, and newborns from HIV‐positive mothers (Figure 3). Among adult patients who presented low NKT‐like cell counts, one third had undergone solid organ (*n* = 9) or haematopoietic cell transplantations (*n* = 32), while among paediatric patients who presented high NKT‐like cell counts, the vast majority had infections (*n* = 95), PFAPA (*n* = 26) or PID (*n* = 21) (Figure 3).

## 4 Discussion

Flow cytometry allows for quickly and accurately identifying imbalances in immune homeostasis, which may also be causes or hallmarks of pathologic conditions, including haematological disorders, infections, and solid tumours [33]. To obtain strength and significance in the results provided, clinical laboratories should rely on robust reference values, difficult to obtain due to the lack of an adequate number of healthy subjects [34, 35]. This limitation becomes more critical concerning immunological parameters, since the immune system considerably changes in relation to age, making it crucial to define specific reference values for narrow age groups, especially for paediatric populations [36].

FACSLyric software provides either percentage or absolute count of NKT‐like cells in immunophenotyping test report, although this data is not yet intended for diagnostic use. Due to the increasing number of works concerning the imbalance of NKT‐like cells in different pathological conditions, for instance in cancer, autoimmunity and infectious diseases [18, 19, 20, 21, 22, 23, 28, 30], it might be useful to include this result in the patient report. This subset is mainly composed of conventional TCRαβ lymphocytes (principally CD8+), but also of unconventional TCRγδ, MAIT cells and iNKT lymphocytes, even if some population cohorts show TCRγδ expression in about half of the population [2, 7, 15, 37, 38].

To date, few works supply the reference values for NKT‐like cells, in particular for paediatric subjects; therefore we have defined our internal normality ranges for this cell population. We found that the NKT‐like cell population increases with ageing, despite the total T lymphocyte count showing an opposite trend that reflects immune system senescence; it has already been proposed that this increase could represent a mechanism to balance the T‐cell reduction [37, 39, 40]. In fact, CD56‐expressing T‐cells are absent in cord blood as well as in newborns, begin to appear in infants, and progressively accumulate throughout life as a consequence of antigenic experience [41].

In our study we used the left‐over blood from selected children outpatients to derive paediatric reference values according to narrow age ranges; in fact, since the enrollment of healthy neonates and children is challenging for ethical reasons, several groups collected left‐over samples from subjects admitted to the hospital for purposes such as surgery, blood grouping, dentistry and so forth [42, 43, 44, 45]. It was challenging to compare our results with other studies on paediatric cohorts. Schatorjé et al. reported absolute reference intervals for NKT‐like cell population, but their limited age range sample size (10–15 subjects/group) made it impossible to determine confidence intervals [36]. Analysing 150 left‐over samples of Thai children divided according to our age stratification, Lerkvaleekul showed data superimposable to ours in children up to 5 years, while in the two subsequent age ranges the values reported were lower [46].

Regarding adult subjects, Melzer and colleagues, in line with our results, evidenced a higher NKT‐like cell count in females, even if in their large cohort of healthy adults they reported higher values than those shown in this study, possibly due to the different age range investigated (40–79 years) [47]. Villegas‐Valverde and co‐workers, using our same lyse‐no‐wash protocol, obtained similar results but with a higher NKT‐like cell count in men than in women [48]. The same gender effect was also described by Andreu‐Ballester, with higher maximum values, probably due to a different management of the outliers [37].

Considering the expression of the T‐cell antigens CD4 and CD8, the fraction responsible for the increase of NKT‐like cell count was the NKT‐like/CD8+, but this is not driven by a parallel increase of cytotoxic T lymphocyte count, as the number of these cells does not undergo substantial modifications with ageing [37, 39, 40, 49, 50]. It can therefore be assumed that the NKT‐like/CD8+ subpopulation could have an important role in the elderly, for example in conditions that can affect them more, such as infections and tumour occurrence, thanks to its mixed NK and T lymphocyte phenotype in addition to the acquisition of innate characteristics [7].

As expected, the pathologic conditions affecting subjects with out‐of‐range NKT‐like cell values were different between the adult and paediatric groups; of note, abnormalities in children were mainly due to an increase in NKT‐like cell count, while adults more frequently had a reduction in these cells. In particular, low counts in adults were often correlated with solid organ or haematopoietic cell transplantations, and thus patients were likely receiving immunosuppressive treatment. Besides, since these cells have a relevant role in Graft‐versus‐Host Disease (GvHD) their monitoring could be important to promptly identify organ‐rejection [51]. Higher values were instead observed in β‐thalassemic patients; in fact, these cells are reported to be an extrinsic factor of thrombotic risk in splenectomized patients [52].

Disorders involving the immune system were the main reason for clinicians to request immunophenotyping, in particular for children prone to prolonged or recurrent infections, for whom the exclusion of an immunodeficiency is essential [53, 54]. Infections, PID and PFAPA were the principal pathologies found in our paediatric subjects having an increased NKT‐like cell count. Consequently, patients with an increased NKT‐like cell count are overrepresented in our paediatric cohorts.

In general, our results are comparable with those described in the literature and fit with the state‐of‐the‐art on NKT‐like cell functions and clinical implications [55, 56].

## 5 Conclusion

In this paper, we defined new reference values for the NKT‐like subpopulation. Notably, the entire diagnostic workup, the reagents and the software employed in the analysis were CE‐IVD certified, which is expected to improve the reproducibility of results and the applicability of the new ranges across different laboratories. We are aware that further studies, involving subjects with different ethnic backgrounds will be needed for the clinical validation of these ranges.

## Author Contributions

Elena Trombetta: conceptualization, formal analysis, writing – original draft; Manuela Liguori: investigation, data curation; Federico Simone Colombo: writing – review and editing; Marta Tornese: investigation, data curation; Alessandra Cattaneo: writing – review and editing; Daniele Prati: resources; Ferruccio Ceriotti: writing – review and editing, funding acquisition; Laura Porretti: conceptualization, writing – original draft, supervision. All authors approved the final version of the manuscript.

## Conflicts of Interest

The authors declare no conflicts of interest.

## Supporting information


**Figure S1:** Flow cytometry gating strategy of NKT‐like cells. (A) Lymphocytes (Lymphs, red dots) were selected on the Side Scatter (SSC) vs. CD45 (PerCP‐Cy5.5) dot plot. Subsequently, NKT‐like cells (CD3+ CD16&CD56+, orange dots) were gated as double‐positive events for CD3 (FITC) and CD16&56 (PE) markers. Absolute counts were obtained using the TruCount tubes (Beads, green dots). CD4 (PE‐Cy7) and CD8 (APC‐Cy7) expression were evaluated on a quadrant plot. (B) Examples of three subject stained with the additional 3‐colour panel composed by CD56 (FITC), CD3 (PE), and CD16 (PE‐Cy7). Total T cells CD3+ (pink dots) were gated on lymphocytes, then CD56 and CD16 expression were evaluated on a quadrant plot.

**Table S1:** Expression of CD4 and CD8 antigens on NKT‐like lymphocytes, expressed as percentages and shown as median with interquartile range (IQR).

## Data Availability

De‐identified patient data used for the results reported in this article, including data in text, tables, and figures, will be available to researchers who provide a methodologically sound proposal to achieve their aims. Proposals should be addressed to elena.trombetta@policlinico.mi.it and laura.porretti@policlinico.mi.it. To gain access, data applicants will need to sign a data access agreement.
