# Supplementary figures and images for: Age‐Matched Reference Values for Circulating Natural Killer T (NKT)‐Like Cells
Source: Scand J Immunol. 2025 Oct 26;102(5):e70062. doi: 10.1111/sji.70062 (PMC12555025; doi:10.1111/sji.70062)

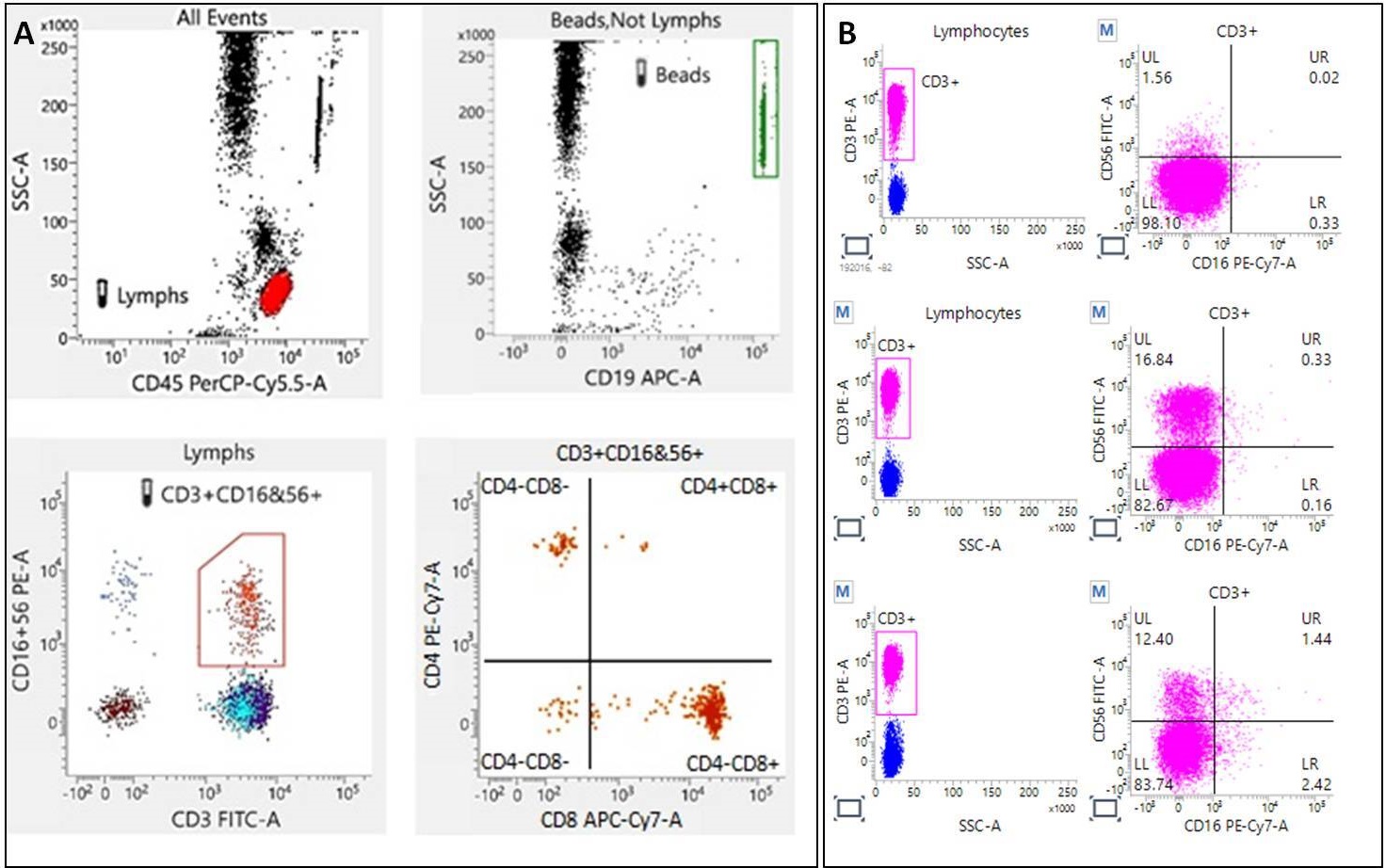

Supplement: Supplementary file 1 — Figure S1: Flow cytometry gating strategy of NKT‐like cells. (A) Lymphocytes (Lymphs, red dots) were selected on the Side Scatter (SSC) vs. CD45 (PerCP‐Cy5.5) dot plot. Subsequently, NKT‐like cells (CD3+ CD16&CD56+, orange dots) were gated as double‐positive events for CD3 (FITC) and CD16&56 (PE) markers. Absolute counts were obtained using the TruCount tubes (Beads, green dots). CD4 (PE‐Cy7) and CD8 (APC‐Cy7) expression were evaluated on a quadrant plot. (B) Examples of three subject stained with the additional 3‐colour panel composed by CD56 (FITC), CD3 (PE), and CD16 (PE‐Cy7). Total T cells CD3+ (pink dots) were gated on lymphocytes, then CD56 and CD16 expression were evaluated on a quadrant plot. [file SJI-102-e70062-s002.jpg]
