# Supplementary material for: Age‐Matched Reference Values for Circulating Natural Killer T (NKT)‐Like Cells
Source: Scand J Immunol. 2025 Oct 26;102(5):e70062. doi: 10.1111/sji.70062 (PMC12555025; doi:10.1111/sji.70062)
Supplement: Supplementary file 2 — Table S1: Expression of CD4 and CD8 antigens on NKT‐like lymphocytes, expressed as percentages and shown as median with interquartile range (IQR). [file SJI-102-e70062-s001.docx]

**Supplemental Table 1.** Expression of CD4 and CD8 antigens on NKT-like lymphocytes, expressed as percentages and shown as median with interquartile range (IQR).

| *Age-ranges* | *0-15 months* | *15-24 months* | *2-5 years* | *5-10 years* | *10-16 years* | *>16 years* | *HD* | *>16 years*  *+ HD* |
| --- | --- | --- | --- | --- | --- | --- | --- | --- |
| *N subjects* | 44 | 72 | 85 | 65 | 66 | 117 | 41 | 158 |
| *%CD4+CD8-* | 36.3 (21-52) | 21.3 (10-39) | 6.1 (3-13) | 7.9 (4-13) | 13.7 (6-21) | 6.7 (3-12) | 7.9 (5-18) | 6.8 (3-14) |
| *%CD4+CD8+* | 14.6 (5-25) | 9.1 (3-16) | 3.9 (0-8) | 3.5 (1-7) | 2.2 (0.7-4) | 1.9 (1-5) | 3.3 (2-8) | 2.4 (1-6) |
| *%CD4-CD8-* | 9.0 (0-14) | 14.3 (6-24) | 23.3 (16-34) | 25.8 (16-34) | 26.6 (16-34) | 16.0 (8-23) | 15.9 (7-29) | 16.0 (7-25) |
| *%CD4-CD8+* | 28.6 (18-45) | 46.5 (29-58) | 54.0 (43-68) | 60.5 (51-68) | 58.8 (51-73) | 72.3 (63-79) | 63.1 (52-78) | 70.7 (60-79) |
